# In Silico Discovery of Antigenic-Secreted Proteins to Diagnostic Human Toxocariasis

**DOI:** 10.1007/s11686-024-00966-0

**Published:** 2025-02-07

**Authors:** María A. Henao, Isabella Cortes, Juan P. Isaza

**Affiliations:** Facultad de Medicina, Grupo Biología de Sistema, Universidad Pontificia Bolivariana, Circular 1a 70-01, Build 11C - 417, Medellín, Colombia

**Keywords:** Antigens, Biomarkers, Immunodiagnosis, Parasitology, Toxocara canis, Toxocariasis

## Abstract

**Background:**

Human toxocariasis is a helminthic zoonosis caused by infection of *Toxocara canis* or *T. cati.* Humans can be infected by through ingestion of embryonated eggs from contaminated water, food or soil. Diagnosis is challenging, immunodiagnosis tests are commonly implemented with major pitfalls in the cross-reactivity with other pathogens, particularly in endemic areas.

**Methods:**

With the aim of identify species-specific genes encoding for highly expressed antigenic proteins, a list of parasites that may infect humans and that might present similar clinical symptoms to *T. canis* infections was built. Only organisms whose genomes were completely sequenced and the proteome predicted were included. First, orthologous proteins were detected and the subcellular localization of *T. canis* proteins was predicted. In order to identify differentially expressed genes encoding proteins in larvae L3, pair-wise comparisons among transcriptomes from body parts and genders were performed. Finally, all secreted proteins classified as species-specific of *T. canis*, whose genes were upregulated in larvae L3 were included in an antigenic prediction.

**Results:**

Twenty-eight parasites were included in the analyses, proteins of *T. canis* were clustered in 11,399 groups, however, 279 were species-specific groups which represent 816 proteins. Three hundred and twenty-two proteins were predicted to be secreted and upregulated in larvae L3, however, after filtering these proteins by their orthology inference, only three proteins met all the features included in this study (species-specific, upregulated, secreted, and antigenic potential). To conclude, our strategy in the study is a rational approach for discovering antigenic proteins to be used in diagnosis.

## Introduction

Human toxocariasis is a helminthic zoonosis caused by infection of *Toxocara canis* or *T. cati*. The adult stage of both species parasitizes the lumen of the small intestine of their definitive hosts, dogs and cats respectively, releasing eggs in feces. Humans can be infected by through ingestion of embryonated eggs from contaminated water, food or soil. Upon the ingestion of eggs, a larval stage hatch from the eggs, and migrates to various tissues, however, larval stages cannot develop into adult worms [1]. In most people, infections are asymptomatic and remains occult, still four clinical syndromes are recognized: visceral larva migrans, ocular larva migrans, cutaneous/covert toxocariasis, and nervous toxocariasis [2].

Diagnosis of human toxocariasis is challenging, mainly because the direct observation through microscopic examination of larvae or the specific detection of larval DNA from tissue or body fluid samples can confirm the infection [3]. Non-specific tests and immunodiagnosis are also implemented. Among non-specific tests are included blood cells count, cytological examination of fluids, and inflammation markers. In immunodiagnosis, a wide variety of assays have been developed. Earliest methods involved soluble extracts from adult worms of *T. canis*, and cultures of *T. canis* larvae allowed to obtain *Toxocara* excretory-secretory (TES) antigens to be used in serological methods [4]. TES antigens have been widely implemented through different approaches (native antigens, recombinant proteins, and chimeric recombinant proteins, among others) with highly variable results in terms of sensibility and specificity. One of the major pitfalls is the cross-reactivity with other pathogens, particularly in endemic areas [4].

The successful discovery of antigens using genomic, transcriptomic and proteomic information has been applied in vaccine development against various types of pathogens [5–7]. This approach, known as reverse vaccinology, in contrast to conventional methods, can significantly reduce the effort, costs and the time required to identify candidate vaccines [8]. This principle can also be applied to the search for candidate antigens for the development of immunodiagnostic assays for different infectious diseases [9, 10]. During the COVID-19 pandemic, Can et al. (2020) used the genome sequence of SARS-CoV-2 and bioinformatic tools to discover antigenic proteins and their epitopes to be used in vaccines or diagnostic methods [9]. In this study, we create a database of proteomes of *T. canis* and infectious organisms commonly included in the differential diagnostic of toxocariasis. Through orthology inference, protein localization, antigenicity prediction and a gene expression profiling, we identify species-specific proteins to reduce cross-reactivity and enhance sensitivity. This strategy led to the identification of three candidate antigens differentially expressed in L3 larvae, with extracellular localization and putative antigenic potential.

## Materials and Methods

A list of parasites that may infect humans and that might present similar clinical symptoms to *Toxocara canis* infections was built. Only organisms whose genomes were completely sequenced and the proteome predicted were included. Table 1 shows all the organisms, the accession codes, and the respective databases included in this study.

**Table 1:** Species included for orthologous inference

Species	Accession code	Database
*Clonorchis sinensis*	PRJNA386618	WormBase Parasite version WBPS16
*Echinococcus granulosus*	PRJEB121	WormBase Parasite version WBPS16
*Fasciola hepática*	PRJEB25283	WormBase Parasite version WBPS16
*Hymenolepis nana*	PRJEB508	WormBase Parasite version WBPS16
*Paragonimus westermani*	PRJNA454344	WormBase Parasite version WBPS16
*Schistosoma mansoni*	PRJEA36577	WormBase Parasite version WBPS16
*Taenia solium*	PRJNA170813	WormBase Parasite version WBPS16
*Hymenolepis diminuta*	PRJEB30942	WormBase Parasite version WBPS16
*Brugia malayi*	PRJNA10729	WormBase Parasite version WBPS16
*Necator americanus*	PRJNA72135	WormBase Parasite version WBPS16
*Strongyloides stercoralis*	PRJEB528	WormBase Parasite version WBPS16
*Trichinella spiralis*	PRJNA12603	WormBase Parasite version WBPS16
*Wuchereria bancrofti*	PRJNA275548	WormBase Parasite version WBPS16
*Ascaris lumbricoides*	PRJEB4950	WormBase Parasite version WBPS16
*Trichuris trichiura*	PRJEB535	WormBase Parasite version WBPS16
*Ancylostoma duodenale*	PRJNA72581	WormBase Parasite version WBPS16
*Angiostrongylus cantonensis*	PRJNA350391	WormBase Parasite version WBPS16
*Toxocara canis*	PRJEB533	WormBase Parasite version WBPS16
*Acanthamoeba castellani*	GCA_000313135.1	AmoebaDB version 53
*Entamoeba histolytica*	GCA_000365475.1	AmoebaDB version 53
*Naegleria fowleri*	GCA_000499105.1	AmoebaDB version 53
*Cryptosporidium hominis*	GCA_002223825.1	CryptoDB version 53
*Cryptosporidium parvum*	GCA_000165345.1	CryptoDB version 53
*Giardia intestinalis*	GCA_000498715.1	GiardiaDB version 53
*Cystoisospora suis*	GCA_002600585.1	ToxoDB version 53
*Toxoplasma gondii*	GCA_000150015.2	ToxoDB version 53
*Leishmania donovani*	GCA_000227135.2	TriTrypDB version 53
*Leishmania infantum*	GCA_900500625.2	TriTrypDB version 53

Orthologous proteins were detected using OrthoMCL v.2.0.9 with default parameters. Briefly, poor-quality proteins were filtered out based on length (minimum length 10 aa) and percent stop codons (maximum percent of stop codons 20). Good-quality proteins were aligned through an all-versus-all BlastP (matrix: BLOSUM62; e-value: 1e-5; Effective size of the database: 359658; Filter query sequence with SEG: yes), and proteins were clustered in orthologous groups using MCL v.14.137 (inflation value: 1.5).

The subcellular localization of *T. canis* proteins was predicted by Phobious v.1.01, SignalP v.5.0b, and TMHMM v2.0c with default parameters. Secreted proteins were defined as proteins with a secretion signal peptide detected by Phobius v.1.01 or SignalP v.5.0b, and without transmembrane regions detected by Phobious v.1.01 and TMHMM v2.0c.

Differentially Expressed Genes (DEGs) in larvae L3 of *T. canis* were identified through pair-wise comparisons among body parts and genders of *T. canis* (L3 vs. female whole worm; L3 vs. male whole worm; L3 vs. female reproductive tract; L3 vs. male reproductive tract; L3 vs. female gut; L3 vs. male gut). Count of aligned reads per gene was obtained from https://parasite.wormbase.org/expression/toxocara_canis_prjeb533/index.html, and edgeR v.3.30.3 was implemented to detect DEGs among comparisons. Because of the gene expression data lacked of replicates, the biological coefficient of variation was arbitrarily set to 0.4. DEGs were considered if the logFC was ≥ 2, the P-value < 0.05 and the false discovery rate was < 0.01.

All secreted proteins classified as species-specific of *T. canis*, whose genes were upregulated in larvae L3 were submitted to the web server VaxiJen v2.0 (http://www.ddg-pharmfac.net/vaxijen/VaxiJen/VaxiJen.html) in order to predict antigenic proteins. Antigenic prediction was conducted with the parasite model and a threshold of 0.6.

**Fig. 1:**
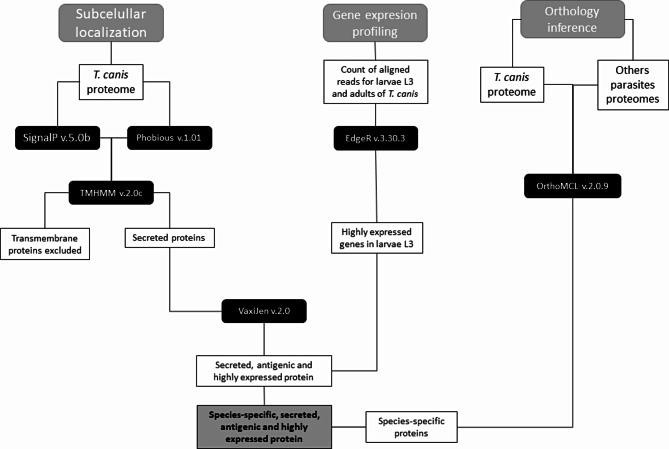
Flowchart summarizing the methodology of the study

Figure 1 describes the analysis pipeline implemented to discover differentially expressed proteins with extracellular localization and putative antigenic potential in L3 larvae of *T. canis*.

## Results

Twenty-eight parasites were included in the analyses, ten Nematoda, seven Platyhelminths, two Amoebozoa, three Discoba, four Alveolate, and one Metamonada (Table 1). A total of 339,454 proteins, comprise the general dataset. Of these, 359,658 good-quality proteins were included for orthologous protein identification, which yielded 49,374 orthologous groups. 20,264 Proteins of *T. canis* were clustered in 11,399 groups, however, 279 were species-specific groups which represent 816 proteins. Singlet proteins were excluded in order to avoid misannotated proteins.

Regarding to the signal peptide prediction, Phobius v.1.01 allowed us to identify 1328 proteins with signal peptide and no transmembrane regions, while SignalP v.5.0b detected Sec signal peptides in 1110 proteins. Considering both results, 1650 of *T. canis* proteins displayed signal peptide, nevertheless, 480 were excluded because TMHMM v2.0c predicted at least one transmembrane helix. Thus, 1170 (5.8%) proteins were classified as secreted proteins.

The gene expression analyses detected transcripts for 19,991 (98.6%) genes of the 20,264 annotated coding sequences. A median of 3091 genes (IQR = 636) were found upregulated in larvae L3 among pairwise comparisons to body parts and genders.

Taking into account the results of the gene expression analyses, and the protein subcellular localization, 322 proteins were predicted to be secreted and upregulated in larvae L3. Among these proteins, 112 (34.8%) exhibited antigenic potential. However, after filtering these proteins by their orthology inference, only 3 proteins met all the features included in this study (specie-specific, upregulated, secreted, and antigenic potential) (Table 2).

**Table 2:** Species-specific proteins with prediction of signal peptide, no transmembrane regions, up-regulation in larvae L3, and antigenic potential

Protein ID^a^	Description	Protein family membership^b^	Paralogous proteins in cluster
TCNE_0000424601-mRNA-1	Unnamed protein product	None predicted	TCNE_0000730501-mRNA-1
TCNE_0000559801-mRNA-1	26 kD secreted antigen (TES-26)	Phosphatidylethanolamine-binding protein	TCNE_0000559901-mRNA-1 TCNE_0000560301-mRNA-1 TCNE_0000560401-mRNA-1
TCNE_0001294601-mRNA-1	Unnamed protein product	Histone H3/CENP-A	TCNE_0001411301-mRNA-1

a: Protein identification code at WormBase Parasite database version WBPS16 (WS279)

b: Based on InterPro 87.0

## Discussion

The diagnostic of parasitic infections relies on laboratory tests accompanied of clinical symptoms. Diagnosis of toxocariasis is challenging, mainly because a direct observation of the parasite through microscopic technics is rarely achieved. For this reason, immunodiagnostic methods are widely used to determine *T. canis* infections. The development of immunological methods has evolved from soluble extracts obtained from adult worms to the implementation of recombinant proteins, showing different levels of sensitivity and specificity [4]. Here we present a rationale selection of antigenic proteins of *T. canis* to be implemented on immunodiagnostic assays.

Our approach depends on the identification of species-specific proteins through an orthology inference, those proteins should present a signal peptide and no transmembrane regions in order to have a higher confidence that they would be released to the extracellular space. In addition, we seek for proteins encoded by highly expressed genes in larvae L3, the infectious stage for humans, because it would produce a high proportion of secreted proteins under normal circumstances and expecting a low posttranscriptional gene expression regulation.

For the orthology inference we included proteomes from 28 parasites that infect humans and present, under some circumstances, similar symptoms to toxocariasis. In this sense, *T. canis* proteins were grouped in 11,399 clusters, a close value to the 10,542 *T. canis* clusters reported by the International Helminth Genomes Consortium in the comparative genome analysis of 91 species. The number of species-specific *T. canis* cluster was also similar, we found 279 clusters containing only *T. canis* proteins, as long as IHGC reported 214 clusters. Differences in the number of clusters could be related to the algorithm used to group proteins and more important to the species included in the analysis. Here we used OrthoMCL v.2.0.9 and proteomes from nematodes, platyhelminths and protozoa parasites, while the IHGC used Ensembl Compara to group proteins from 81 platyhelminths and nematodes (parasitic and free-living) and ten outgroups from other animal phyla [11].

Zhu et al. (2015) reported that the *T. canis* secretome comprise at least 870 proteins with diverse functions, our analyses detected 1170 proteins. The difference between predictions is associated to the gene annotation in the *T. canis* genome projects. Zhu et al. predicted 18,596 proteins in the PN_DK_2014 strain [12], while the International Helminth Genomes Consortium predicted 20,264 proteins for the Brazil/Equador strain [11]. Our prediction represents approximately 1.2% more secreted proteins.

Our gene expression analysis identified a median of 3091 (IQR = 636) DEGs among comparisons of larvae L3 and individual body parts of male or female adults. A similar number of DEGs were observed by Zhu et al. (2015), they described 2833 L3-overexpressed genes compared to adults that were related to the metabolic pathways Neuronal signalling and Cuticle formation/shedding [12]. It is important to mention that both gene expression analyses, Zhu’s and ours, were carried out with the same dataset of reads.

The rational selection implemented in the present study, led us to identify three antigens: histone H3, TES-26, and an unnamed protein product.

Eukaryotes express different variants of the histone H3. These variants have a well-conserved globular C-terminal domain and a more divergent and unstructured N-terminal domain. Usually H3 variants have a nuclear localization, where the C-terminal domain is involved in the histone-histone interaction at nucleosomes, and the N-terminal domain is the target of post-translational modifications [13]. However, histones not only fulfill a nuclear function but can also be translocated to the extracellular space, acting as endogenous danger signals or DAMPs in human cells [14]. Hyperacetylated histone H3.3 is resistant to proteasomal degradation, which causes its accumulation in the extracellular space [15]. It has been demonstrated that histones exhibit significant proinflammatory and cytotoxic activity [16], and their levels increase under conditions of cellular stress [14], cancer [17], infection [18], and presence of parasites [19]. We predicted an extracellular localization for the histone H3 variant of *T. canis*, one of the three discovered proteins that are specie-specific, upregulated, secreted, and possess antigenic potential. Marcia et al. (2018) through a proteomic approach in *T. canis*, identified 64 secreted proteins, and the histone H3 was among the most abundant secreted proteins, which confirm our results as a secreted protein with high expression levels in larvae L3 [20]. Others proteomic strategies had demonstrated the extracellular localization for histones of fungal pathogens and protozoa parasites such as *Cryptococcus neoformans*, *Histoplasma capsulatum* (Hc), *Plasmodium falciparum*, *Toxoplasma gondii* and *Leishmania donovani* [21–24]. Specifically in Hc, an histone-like protein with antigen functions was identified and suggested as a potential candidate for vaccine development [25]. Similarly, in *Leishmania infantum*, histone H3 was described as an immunodominant antigen in canine visceral leishmaniasis [26], with the immunogenic potential related to the first 40 amino-acid residues of the N-terminal domain [27].

TES-26 is found in *T. canis* larval stages, but not in the adult stage of the parasite. It consists in a 262 amino acids surface protein, that belongs to the phosphatidylethanolamine binding proteins (PEBP) family and its high immunological specificity reliefs in the low molecular weight of 28 kDa including signal peptide or the name giving 26 kDa molecular weight without it [28]. It is involved in lipid binding and the inhibition of inflammatory cell signaling [20]. Although TES-26 is mostly hydrophilic, it contains a hydrophobic region composed by two serine trypsin-like metalloproteinases domains, Shk1 and ShK2, these two domains are involved in the modulation of inflammatory signaling pathways such as MAPK3 and NF-kB [20, 29]; the combined action of both domains is of major importance for the worms’ survival inside the host, which is unusually prolonged for a nematode [30]. In other organisms, like *Stociactis heliantus a* marine anemone, ShKs proteins are described as potassium channel toxin inhibitors [31]. In previous studies have been proved that TES immune evasion mechanisms could imply the protein coating the parasites integument glycoproteins after being detected by specific antibodies, then forming a negatively charged “fuzzy coat” and allowing eosinophils to adhere, finally, such “fuzzy coat” is released and the larvae escapes the immune response [20, 30–32].

TES-26 has been implemented as antigen for human toxocariasis diagnostic since its discovery by Gems et al. (1995), even a poor diagnostic value was found (11.5%) when it was tested with serum from toxocariasis patients through Western blot [28]. However, in a Luminex bead-based assay, Anderson et al. (2015) reported a sensitivity and specificity higher, 85% and 91%, respectively [33]. Mohamad et al. (2009) found a specificity of 96.2% and a sensitivity of 80%, through an IgG4 ELISA assay [34]. Immune potential increases as it merges several aspects meant to facilitate the assertion of a diagnostic. Hence, TES-26 remains quite promisingly attainable of the fact that B cells present two principal epitopes able to recognize said protein: QPSTPAA and LYNLVVQD. Furthermore, 19 B-cell antigenic epitopes in TES-26 have already been identified, contributing valuable information to the development of immunoassays [29]. Organisms with protein homologues to TES-26 include fatty acid binding proteins (FABPs) of *Fasciola hepatica*, FABP homologue As-p18 of *Onchocerca volvulus* and *Schistosoma mansoni* Sm14, related to cross immunological protection against schistosomiasis, TES-26 could represent a good antigen candidate [20].

Finally, we identified as a potential antigen an uncharacterized protein of 114 amino-acid residues with a theoretical molecular weight of 12.3 kDa. This protein is encoded by a transcript with two exons. Sequence search for functional domains did not display successful results. So, little information is available for this protein and more studies are required to characterize its function. Investigation of this uncharacterized protein represents a more complex matter, because of the general lack of annotation of the worm’s proteins, ES pathways, experimental identification, among others.

The early detection and diagnosis of *T. canis* facilitate prompt and accurate intervention. Therefore, the identification of specie-specific antigens is crucial. The three proteins identified, which were demonstrated to be species-specific through orthologous inference, secreted without transmembrane regions, overexpressed, and detected by the immune system, are potential new immunogenic agents and biomarkers. These proteins could be applicable for pharmacological purposes, diagnostics, and even vaccine development. The identified proteins could significantly reduce cross-reactivity compared to other diagnostic methods. The strategy used shares similarities with other antigen and protein identifications in *Mycoplasma bovis* [35], *Entamoeba histolytica* [36], *Trichuris trichiura* [37], *Toxoplasma gondii* [38], and *Taenia solium* [39].

## Conclusions

Our study identified three species-specific antigens: histone H3, TES-26, and an uncharacterized protein, derived from *Toxocara canis*, which hold significant potential for improving the diagnosis of toxocariasis. By targeting these specific proteins, we propose a more precise and reliable method for immunodiagnostic tests that can reduce cross-reactivity and enhance diagnostic accuracy, thereby minimizing errors and confusion with similar clinical diseases. This advancement is crucial for timely and appropriate treatment interventions, ultimately leading to better patient outcomes and more effective disease management. However, further investigation is required to validate the efficacy of these proteins in clinical settings and to fully integrate these findings into routine diagnostic practice.

## Data Availability

No datasets were generated or analysed during the current study.
